# Performance of TiO_2_/UV-LED-Based Processes for Degradation of Pharmaceuticals: Effect of Matrix Composition and Process Variables

**DOI:** 10.3390/nano12020295

**Published:** 2022-01-17

**Authors:** Danilo Bertagna Silva, Gianluigi Buttiglieri, Bruna Babić, Danijela Ašperger, Sandra Babić

**Affiliations:** 1 Faculty of Chemical Engineering and Technology, University of Zagreb, Trg Marka Marulića 19, 10000 Zagreb, Croatia; dsilva@fkit.hr (D.B.S.); bbabic@fkit.hr (B.B.); diva@fkit.hr (D.A.); 2 Catalan Institute for Water Research (ICRA-CERCA), C. Emili Grahit, 101, 17003 Girona, Spain; gbuttiglieri@icra.cat; 3 Universitat de Girona, Girona, Spain

**Keywords:** light-emitting diode, TiO_2_ nanofilm, photocatalysis, design of experiments, advanced oxidation processes, contaminants of emerging concern

## Abstract

Ultra-violet light-emitting diode (UV-LED)-based processes for water treatment have shown the potential to surpass the hurdles that prevent the adoption of photocatalysis at a large scale due to UV-LEDs’ unique features and design flexibility. In this work, the degradation of five EU Watch List 2020/1161 pharmaceutical compounds was comprehensively investigated. Initially, the UV-A and UV-C photolytic and photocatalytic degradation of individual compounds and their mixtures were explored. A design of experiments (DoE) approach was used to quantify the effects of numerous variables on the compounds’ degradation rate constant, total organic carbon abatement, and toxicity. The reaction mechanisms of UV-A photocatalysis were investigated by adding different radical scavengers to the mix. The influence of the initial pH was tested and a second DoE helped evaluate the impact of matrix constituents on degradation rates during UV-A photocatalysis. The results showed that each compound had widely different responses to each treatment/scenario, meaning that the optimized design will depend on matrix composition, target pollutant reactivity, and required effluent standards. Each situation should be analyzed individually with care. The levels of the electrical energy per order are still unfeasible for practical applications, but LEDs of lower wavelengths (UV-C) are now approaching UV-A performance levels.

## 1. Introduction

Water scarcity across the globe demands an effort to find efficient and sustainable treatments that will allow its safe reuse [1,2]. Furthermore, pharmaceutical active compounds (PhACs) are commonly found in wastewater treatment plant outlets, since some of them are resistant to conventional physical and/or biological treatments [3,4,5,6]. PhACs may then end up in waterbodies, persist in the environment and bio-accumulate, causing diverse sorts of endocrine disruptions in aquatic life, even at very small concentrations (ng/L, µg/L) [7,8]. Studies have shown that their effects on human health are highly unpredictable given their chemical diversity and the numerous interactions they can have with other substances present in water [9].

Among possible water treatment solutions, advanced oxidation processes (AOPs) have shown a high degradation potential due to the formation of reactive oxygen species (ROS), especially the hydroxyl radical (^•^OH), which are able to oxidize persistent pollutants [10,11,12]. However, these processes are highly energy-demanding [13]. Photocatalysis using TiO_2_ has been investigated in depth in previous decades due to its capacity for generating radicals in water using only light—be it solar or artificial—but real-life applications have rarely been adopted due to the process’ low photonic efficiency, the concerns regarding additional separation steps (for TiO_2_ as a powder), mass transportation issues, and lower degradation rates (for immobilized TiO_2_) [14,15,16]. Other reasons for the large number of works dealing with TiO_2_ photocatalysis are its various enhancement possibilities. Radical formation can be increased by adding H_2_O_2_ to matrices [17], altering TiO_2_’s surface by doping or heterojuction coupling techniques which increase the catalyst’s photoactivity in the solar range [18,19]. The photocatalytic potential of other materials besides TiO_2_ has also been investigated [20]. The advantages of photocatalysis over other AOPs are the possibility of generating not only the hydroxyl radical but other reactive species, such as the superoxide radical (^•^O_2_^–^) and positive holes (*h*^+^), allowing different reaction routes [15].

To reach an in-depth understanding of the variables which influence photolysis and photocatalysis, a critical analysis of performed experiments should take place. Works focusing on the degradation of single spiked target PhACs in ultra-pure water are ubiquitous [20,21,22,23,24], as are investigations of radical scavengers, which are responsible for degradation hindering [16,25,26,27], though mixtures of compounds are seldom considered. More recently, the concern about optimizing energy expenses has established the electrical energy per order (*E_EO_*) as a key parameter to compare different AOPs [28,29,30,31]. *E_EO_* is defined as the amount of energy necessary to remove 90% of a target pollutant in a fixed volume of water (generally expressed as kWh m^−3^).

The development of ultra-violet light-emitting diodes (UV-LEDs) and recent advances in their wall-plug efficiency and affordability has opened up new possibilities for photocatalysis, since many features of UV-LEDs give them an upper hand in comparison to traditional low- or middle-pressure Hg UV lamps [32]. LEDs are built from durable ceramic materials, are mercury-free, have longer lifetimes, and have no required warm-up periods (which allows the use of photonic efficiency techniques, such as controlled periodic illumination (CPI)) [33]. Their dimensions can be approximated as point sources, allowing very flexible design and small-scale point of use applications [32,34,35]. Previous studies highlighted the fact that the development of UV-LEDs is still very recent, so the technology is at an early stage [35,36]. Lower external quantum and wall-plug efficiencies, especially for lower wavelengths, were always considered the main hurdle for further adoption of UV-LEDs in the last decade [33]. Nevertheless, a recent review shows that *E_EO_* values of UV-LEDs and Hg UV mercury lamps are on the same order of magnitude [32] and comparisons between them are now feasible.

A few studies have engaged in designing and evaluating innovative UV-LED photoreactor designs [37,38,39], and some of them have applied design of experiments (DoE), a powerful tool to optimize highly complex systems and find optimal conditions [40,41,42]. Studies evaluating the performance of UV-LED photocatalytic processes in real matrices, such as tap water, river water, or wastewater at real pollutant concentrations, are scarce [8,23,40,43,44]. The LED-exclusive feature of CPI has only been investigated for photocatalysis in simple scenarios; in MilliQ (MQ) water and rudimental reactor designs. The simultaneous use of light sources of different wavelengths, which is possible due to the point-source character of LEDs, is another field that has not been explored by scientists and engineers. Additionally, simultaneous photocatalytic degradation studies of multiple PhACs in water (more than three) often demand powerful analytical techniques and complex chromatographic methods; therefore, more studies on the topic are necessary to further the development of this technology.

Additionally, it is paramount that awareness about the issue of PhACs and other contaminants of emerging concern keeps growing among the general population, which would press for more strict water regulations across the world and subsequently generate more interest in the further development and adoption of AOPs by water technology specialists.

The goal of this work was to investigate the simultaneous degradation of five different PhACs by UV-LED-based processes in a lab-scale reactor under a wide range of different scenarios. The chosen PhACs were ciprofloxacin (CIP), sulfamethoxazole (SMX), trimethoprim (TMP), venlafaxine (VX), and desmethylvenlafaxine (DV)—all currently on the 2020/1161 EU Watch List of substances to be monitored [45]. The degradation mechanisms of each of them individually and in a mixture were compared. Two different DoEs were applied. The first DoE investigated the influence of different water matrices (MQ and tap water), LED wavelengths (UV-C and UV-A), the presence or absence of TiO_2_ nanofilm, and controlled periodic illumination (CPI) on PhAC degradation. The second DoE studied the impact of matrix components (bicarbonates, nitrates, and humic acids) on each of the PhACs’ apparent first-order degradation rate constant (*k_app_*) during UV-A photocatalysis. The impact of initial pHs was also evaluated. Furthermore, the estimation of *E_EO_* values related to different photoreactor designs and simultaneous wavelengths, while targeting PhAC removal, enriched the discussion.

The main contribution of this work is a complex analysis which simultaneously encompasses matrix composition, photoreactor parameters, and recently developed LED-exclusive features in relation to the photocatalytic degradation of contaminants of emerging concern, as well as the impact on degradation kinetics, energy consumption, and effluent toxicity.

## 2. Materials and Methods

### 2.1. Chemicals and Reagents

High purity (>98%) analytical standards of CIP (CAS no. 85721-33-1), SMX (CAS no. 723-46-6), TMP (CAS no. 738-70-5), and humic acids (CAS no. 1415-95-6) were supplied from Sigma-Aldrich (St. Louis, MO, USA). VX (CAS 99300-78-4) and DV (CAS no. 93413-62-8) were supplied by Tokyo Chemical Industry Co. LTD (Tokyo, Japan). Triethanolamine (CAS no. 102-71-6) was supplied from Carlo Erba Reagents (Milan, Italy). Ammonium oxalate (CAS no. 6009-70-7), sodium nitrate (CAS no. 7631-99-4), and sodium bicarbonate (CAS no. 144-55-8) were supplied by Kemika (Zagreb, Croatia). Acetonitrile was HPLC grade (J. T. Baker, Deventer, Netherlands).

### 2.2. Water Matrices

The photolytic and photocatalytic degradation of selected PhACs were investigated in two water matrices: ultrapure water and tap water. MQ water (pH = 5.8) was prepared by the Millipore Simplicity UV system (Millipore Corporation, Billerica, MA, USA). Tap water was sampled at the laboratory faucet at the Faculty of Chemical Engineering and Technology, University of Zagreb. Prior to the sampling, the faucet was turned on and left to run at a uniform rate to flush standing water from the service pipes (2–3 min). Tap water was analyzed for pH, total organic carbon (TOC), and inorganic ion content. Table S1 in the supplementary information shows the composition of tap water.

### 2.3. Experimental Set-Up

Two identical cylindrical quartz reaction vessels with an inner diameter of 37 mm, length = 150 mm and wall thickness = 1.5 mm were adopted. In one of them, nanostructured TiO_2_ film was immobilized on its inner sidewall by the sol–gel method and dip-coating technique. For the preparation of colloidal TiO_2_ solution (sol), the following components were used: titanium(IV) isopropoxide (Ti(C_3_H_5_O_12_)_4_)—TIP as a precursor; i-propanol (C_3_H_7_OH)—PrOH as a solvent; acetylacetone (CH_3_(CO)CH_2_(CO)CH_3_)—AcAc as a chelating agent; nitric acid (HNO_3_)—HN-0.5 M as a catalyst. The molar ratio of these reactants was: TIP:PrOH:AcAc:HN = 1:35:0.63:0.015. The sol was poured into the cylindrical reactor, kept there for 10 min, and slowly poured out. After that, the film was dried at 100 °C for 1 h prior to the deposition of the next layer. Following the deposition of the three layers, the film was annealed at 550 °C for 4 h. The preparation of the nanofilm, its characterization (the crystalline structure, crystalline phases composition, the surface topography, the roughness of the TiO_2_ film, and the grain size distribution of the TiO_2_ film), and immobilization are described in detail in [46,47].

A schematic drawing of the experimental set-up is shown in Figures S1 and S2. Six UV-LED strips were attached to the support as external vertical columns of diameter = 60 mm. The light sources were all facing towards the cylinder’s central axis and their distribution was radially symmetric, with intervals of 60°. Using the control board, it was possible to turn on all six LED columns simultaneously or half of them (just UV-A or just UV-C). Each LED column had 125 mm of height. The UV-A strip contained 15 LED sources, spaced vertically by 8.3 mm, while the UV-C strip contained eight LED sources, spaced vertically by 16.6 mm. A UV-A strip was always positioned between two UV-C strips. Each column could be easily (dis)attached to the system, so at any moment it was possible to decide how many UV-C and UV-A strips were illuminating the reaction. A detailed description of the experimental set-up can be found in the previous work by [38].

UV-LED strips in the UV-A range (365 nm) and UV-C range (272 nm) were provided by Waveform Lighting (Vancouver, WA, USA). Photometric specifications, emission spectrum, dimensions, and other data are available in the product’s specification datasheet [48,49] (summarized in Table S2). The LED strips were connected to an Arduino Pro Mini microcontroller coupled with IRFZ44 N MOS-FETs. It was possible to control the duty cycle of the LEDs using the pulse-width modulation script on the Arduino that was 490 Hz [50]. The output power of the system was measured by the +UT230B power meter by UNI-Trend Technology (Dongguan, China).

All experiments were performed in a dark room. The temperature of the reaction solution remained at (21.0 ± 2.0) °C throughout the experiments.

The list of acronyms used for the experimental set-ups can be found in Table S3.

### 2.4. Analytical Determination

To determine the degradation rates of CIP, TMP, SMX, VX, and DV, the samples were directly analyzed by HPLC-PDA (Waters 2795 Alliance HPLC System with 2996 PDA-Detector) and Masslynx software provided by Waters (Milforn, MA, USA. The separation was carried out with a Kinetex C18 column (150 mm × 4.6 mm, 5 mm, 100 Å, Phenomenex). The mobile phase was composed of 0.1% formic acid in ultrapure water (A) and 0.1% formic acid in acetonitrile (B). The initial volume proportion of eluents was A:B = 89:11 (*v*/*v*) until *t* = 13.5 min. From that point until *t* = 15.0, linear gradient elution was applied and the proportion of A:B = 83:17 (*v*/*v*) was achieved, which was kept constant until *t* = 25 min. At this point, the mobile phase composition had a step change back to the initial one (11% B) until the end of elution, at *t* = 28 min. The flow rate was 1.0 mL min^−1^. The column temperature was 20 °C. The volume analyzed for each sample was 20 μL. CIP, TMP, SMX, VX, and DV were detected at the wavelength of 278.8 nm, 273.8 nm, 269.8 nm, 274.8 nm, and 274.8 nm, respectively. The retention time of each compound (in minutes) was 11.3 (CIP), 6.3 (TMP), 19.4 (SMX), 23.0 (VX), and 9.6 (DV). The calibration curve was linear between 0.2 mg L^−1^ and 2 mg L^−1^, with *R*^2^ > 0.995.

Toxicity tests were performed according to the standard bioluminescent method described in ISO 11348-3:2007 standard with fresh *Vibrio fischeri* bacteria. Lyophilized bacteria were obtained from Hach Lange (luminescent bacteria test LCK 484, Dusseldorf, Germany). Bacterial luminescence measurements were performed on a LUMIStox 300 Hach Lange instrument (Dusseldorf, Germany) with a thermostated LUMIStherm block for incubation of bacteria. Luminescence was monitored initially and after 30 min as a parameter indicating toxicity or inhibition. All measurements were performed at an instrument operating at a temperature of (15 ± 1) °C.

Total organic carbon (TOC) was measured by the TOC analyzer, type TOC-VCPH, Shimadzu Co. (Kyoto, Japan). [51].

### 2.5. Design of Experiments

Two DoEs were prepared using Design Expert software (version 12), Stat-Ease, Minneapolis, MN, USA). In the first DoE, the full factorial design (2^4^ = 16 experiments) with categorical variables was used to investigate the effect of process variables and matrices on PhAC degradation efficiency. Table 1 shows its independent variables. The dependent variables were the *k_app_* for each of the five PhACs, TOC removal (after 60 min of illumination time), and the Δ luminescence (difference between final and initial *Vibrio fischeri* luminescence). Pareto charts representing the significance of each effect were obtained. The initial concentration of each PhAC in the mixture was 2 mg/L. Although this value is higher than the range typically found in waterbodies [7], it was adopted for easier monitoring and easier evaluation of the influence of the investigated parameters.

The purpose of DoE is to obtain an empirical mathematical model to predict the outcome of a dependent variable in reference to a group of independent variables and to quantify the significance of each of these independent variables (or their combination) to the outcome. The relationship between response *Y* and the independent coded parameters *X_i_* could be estimated by the first order polynomial model, as shown in Equation (3). It must be stated that, when working with categorical variables (e.g., presence or absence of catalyst, matrix as MQ water or tap water), Equation (1) is not valid for any coded values except −1 and +1.

(1) $$\begin{array}{ccc} Y=\beta_{0}+\beta_{1}X_{1} & +\beta_{2}X_{2}+\beta_{3}X_{3}+\beta_{4}X_{4}+\beta_{5}X_{1}X_{2}+\beta_{6}X_{1}X_{3}+\beta_{7}X_{1}X_{4}+\beta_{8}X_{2}X_{3}+\beta_{9}X_{2}X_{4} & +\beta_{10}X_{3}X_{4}\\ & +\beta_{11}X_{1}X_{2}X_{3}+\beta_{12}X_{1}X_{2}X_{4}+\beta_{13}X_{1}X_{3}X_{4}+\beta_{14}X_{2}X_{3}X_{4}+\beta_{15}X_{1}X_{2}X_{3}X_{4}\; & \end{array}$$

In this study, the dependent variables are the first order apparent degradation constant rate (*k_app_*) for each PhAC, the abatement percentage of total organic carbon (%TOC decrease), and the difference between final and initial *Vibrio fischeri* luminescence of the effluent (Δ luminescence,) representing toxicity. The independent variables and their range of study are arbitrarily chosen based on what the authors want to investigate (see Table 1 for the independent variables and their tested range). A full factorial design with two levels requires experimental data of all possible combinations of independent variables in their minimum and maximum range (coded values −1 and +1). For four variables, this demands 4^2^ = 16 experiments. Each one of the *β*_i_ coefficients obtained represents the intensity of the effect of each independent variable (or their combination), as well as if the latter will increase (positive effect) or decrease (negative effect) the dependent output. A positive effect for combined variables means that either the simultaneous increase or decrease of both variables will increase the outcome of the dependent variable (because the result of multiplying two positive or two negative numbers is positive), while increasing one of the independent variable’s coded values and reducing the other will result in a decrease of the outcome of the dependent variable (because the product of a positive and a negative number is negative). The Design Expert software provides all *β*_i_ coefficients side by side in a Pareto chart. The effects beyond the *t*-value limit are considered significant by an analysis of variance (ANOVA) with 95% confidence and are included in the final model. Effects beyond the Bonferroni limit are strongly statistically significant corrected for multiple testing.

A second DoE was performed to investigate the effect of matrix components on the degradation rate constants of investigated PhACs. A randomized response surface Box–Behnken design with 16 runs was made with three independent variables on three levels (Table 2). The obtained *F*-values, representing the significance of each effect, were analyzed. The software also provided surface graphs of the system and the ANOVA analysis of coefficients.

The concentration ranges of bicarbonates and nitrates were loosely based on their common concentration in tap water, as well as their regulation limits (50 mg/L for nitrates) [52]. As to humic acid, the range of values was arbitrarily set to simulate the effect of the presence of organic matter in the matrix. The *k_app_* of each of the five PhACs was set as the dependent variable. UV-A photocatalysis experiments were performed with the original mixture of five PhACs in MQ water. Choices of square root transformations and different fits (linear or quadratic) were made to increase the adjusted and predicted *R*^2^ values. The initial concentration of each PhAC in the mixture was 2 mg/L.

### 2.6. Electrical Energy per Order (E_EO_) Analysis

*E_EO_* is a figure of merit defined by [53] as the amount of energy (kWh m^−3^) required to degrade 90% of a target pollutant in 1 m^3^ of water [54]. It can be obtained via Equation (2) [53], in which *t* is the amount of time (min) required to reach 90% degradation, *P* is the power output of the system (W), and *V* is the reaction volume (L) of the system [22,29,55].

(2) $$E_{EO}=\frac{P\;\cdot t}{V\cdot 60}$$

Following the same deduction from a previous work [38], the *E_EO_* can be calculated for each PhAC using Equation (3):

(3) $$E_{EO}{\left({\mathrm{kWhm}}^{-3}\right)}_{PhAC}=\frac{-ln0.1}{k_{app}\;{\left(\min^{-1}\right)}_{PhAC}}\cdot \frac{P\;\left(W\right)}{0.14\;\left(L\right)\cdot 60}$$

*P* is obtained from the power meter read. The average reactor volume during the experiments is *V* = 0.140 L, considering the volume variation caused by sample collection. When the kinetic constant *k_app_* (min^−1^) is obtained, it is possible to calculate *t* for 90% of degradation (*C*/*C*_0_ = 0.1) by solving the first order kinetic equation (ln(*C*/*C*_0_) = *k_app_*·*t*). When no reaction takes place (*k_app_* = 0) for a given PhAC, *E_EO_* is theoretically infinite.

## 3. Results and Discussion

### 3.1. PhAC Degradation: Individually and in a Mixture

**Figure 1:**
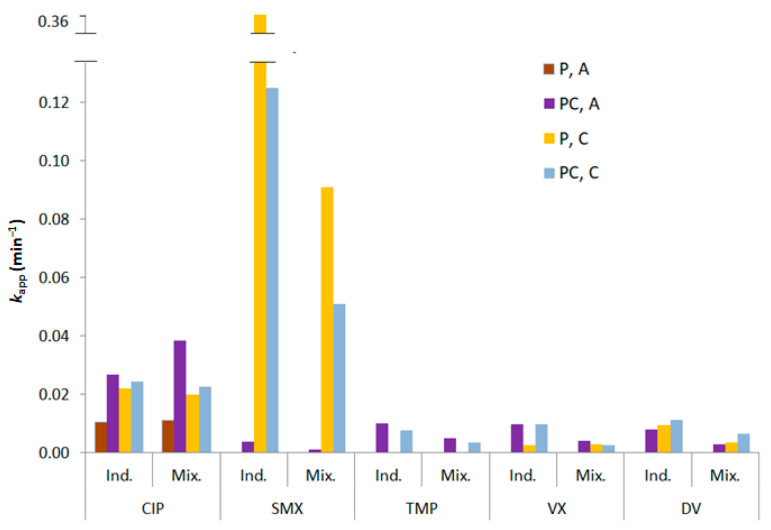
*k_app_* values for PhAC degradation individually (Ind.) and in a mixture (Mix.) in MQ water and at a concentration of 2 mg/L.

Figure 1 shows the *k_app_* values for the degradation of the five PhACs tested individually and in a mixture, in MQ water. Figure S3 shows degradation profiles for the individual degradation experiments. It can be observed how the pollutants were degraded differently in the treatments. The degradation depends on: (1) the types and amount of available oxidative species; (2) the PhACs’ respective reactivity with each of the generated oxidative species [5]; (3) the interaction between the PhACs and the catalyst surface (adsorption) [56]; (4) the ionic state of each PhAC in solution (there might be distinct reactivity for the same parent compound for both photolytic and photocatalytic routes); (5) PhAC absorption spectrum and quantum yield for a given wavelength (specific for photolysis) [57,58]. Most of these points depend, directly or indirectly, on the matrix composition and pH [59].

The only compound degraded by UV-A photolysis was CIP. The fastest degradation was for SMX during UV-C photolysis, attaining better *k_app_* results without the catalyst. A possible explanation is that TiO_2_ nanofilm may cause a screening effect, given that UV-C light has lower penetration capacities than UV-A [60]. Since UV-C photolytic degradation for SMX was already very fast (complete degradation in less than 45 min in the individual test), the presence of the TiO_2_ nanofilm hinders the direct contact between SMX and photons, and the increase in degradation provided by ROS production does not compensate for the losses in available photons for the direct photolysis faster degradation route [61,62]. TMP, VX, and DV had lower degradation rates overall, with *k_app_* values below 0.01 min^−1^. TMP was completely impervious to both UV-A and UV-C photolysis.

When considering PhAC mixtures, the *k_app_* of four compounds (SMX, TMP, VX, DV) decreased by an average of 50%. This is expected considering that a higher amount of pollutants lowers the availability of degradation agents, such as radicals and photons [33,63]. Conversely, a 40% increase in UV-A degradation of CIP was observed in the presence of the catalyst when CIP was mixed with the other compounds. This behavior can be related to the solution pH of 6.0 in the case of CIP-only experiments and of 6.7 in the mixture (Table S4, experiments 1–20 for individual PhACs and 24–27 for PhACs mixture). In fact, the ionization state under a given pH should be considered. CIP has a p*K*_a_ of 6.08 [63], predominantly being cationic at pH below that value and neutral above it until pH = 8.4 [44,63]. The ionization state interferes with the reactivity of the molecules with photons and radicals, as well as the adsorption rates at catalysts’ surfaces and, hence, it can be related to photolytic and photocatalytic degradation rates [64]. The cationic state of CIP is reported to have lower degradation rates for both photolysis and photocatalysis [63], and since the ionic state shifts from cationic to neutral around the tested pH range (Table S3), this can explain the *k_app_* increase. The p*K*_a_ values of TMP, SMX, VX, and DV are 7.1 [64], 5.6 [65], 9.0 [66], and 9.0 [67], respectively. Among these, the only compound with a shift of ionic states in the tested range was SMX. The fact that this compound is depleted faster in its neutral state (pH < 5.6) [65] further explains why its *k_app_* was so high in the individual experiments.

TiO_2_′s point of zero charge is 6.3 [68], and the strength of its interaction with each molecule will depend on the ionic state of both the molecule and the surface of TiO_2_. Although the affinity between the PhACs and TiO_2_ surfaces is a relevant parameter for photocatalytic routes, the catalyst surface area was much smaller than in the case of porous catalysts given that immobilized TiO_2_ was used in this research [14]. Thus, the adsorption mechanisms were not as prominent as the ones taking place in the bulk of the solution because even if the surfaces attract each other strongly, the available area of the catalytic site was still small. This being so, when immobilized catalysts are used, the ionization state can play a bigger role in the photolytic rates [22,69,70]. Since photolysis takes place in the bulk of the reactor, it does not rely on adsorption phenomena and it is not limited by mass transport [71]. For photocatalytic routes with immobilized catalysts, the prominence of other factors, such as the amount and type of generated ROS, can be of more relevance.

### 3.2. The Effect of the Catalyst, Light Wavelength, Controlled Periodic Illumination, and Matrix

A full factorial design investigated the effect of process variables on the efficiency of PhAC photolytic and photocatalytic degradation and the degradation rate constant; the %TOC decrease and the Δ luminescence were evaluated.

#### 3.2.1. *k_app_* Analysis

The list of experiments and the obtained results (*k_app_*) for the full factorial design are presented in Table S4 (experiments 24–39). Based on these data, the coded Equations (models) (4)–(8) were obtained for each of the five PhACs by selecting the effects with *p*-value > 0.05 and those necessary by the hierarchy, as calculated by the software (Design Expert 12).

(4) $$k_{app}{}_{\left(CIP\right)}=0.0317+0.0046A-0.0020B+0.0041C-0.0033D-0.0044AB-0.0053AC+0.0086BC-0.0020CD\;$$

(5) $$k_{app}{}_{\left(SMX\right)}=0.0225-0.0122A-0.0182B-0.0034C+0.0159AB+0.0053AC+0.0077BC$$

(6) $$k_{app}{}_{\left(TMP\right)}=0.0013-0.0008A+0.0004B+0.0010C+0.0002AB-0.0005AC+0.0007BC$$

(7) $$\sqrt{k_{app}{}_{\left(VX\right)}}=0.0368-0.0097A-0.0060B+0.0235C+0.0059AB+0.00359809AC+0.0074BC+-0.0076BC$$

(8) $$\sqrt{k_{app}{}_{\left(DV\right)}\;}=0.0408-0.0111A-0.0202B+0.01157C-0.0100AC+0.0091BC$$

**Figure 2:**
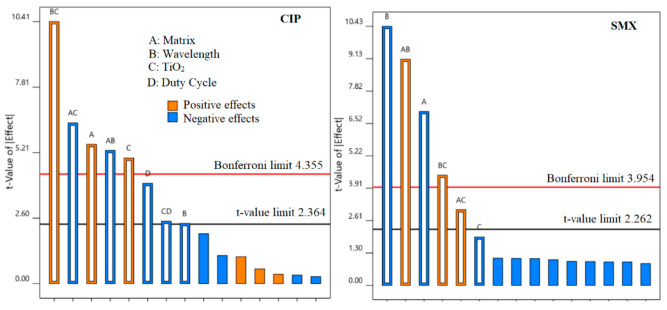

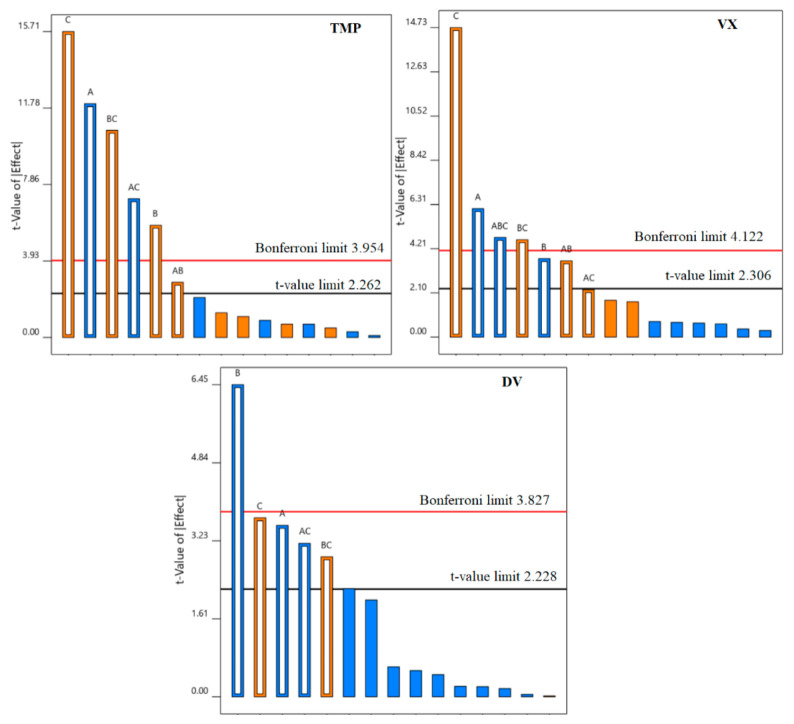
Pareto charts and significant effects (above Bonferroni limit) for *k_app_*.

Figure S4 shows the ANOVA results provided by the software, confirming that all obtained models are all significant. The Pareto charts for all the compounds are shown in Figure 2.

BC, significant for all PhACs, represents the combined impact of wavelength (B) and catalyst presence (C). The best results were obtained with the catalyst combined with UV-A or when no catalyst was used in the presence of UV-C. This is explained by the fact that, on the one hand, UV-A rays were not able to degrade the tested PhACs by means of photolysis alone (except CIP). On the other hand, UV-C rays are highly capable of photolytic degradation but suffer from a strong screening effect in the presence of the catalyst. The overall results indicate that the enhancement obtained by the presence of the catalyst, when UV-C was used, did not compensate for the photo absorption losses by the system.

The influence of the matrix (A) was significant in all the cases. Tap water contains many ions (Table S1) which can influence the degradation positively or negatively [16]. The difference in the pH between tap and MQ water can also impact the degradation rates [63]. All compounds degraded slower in tap water due to the presence of scavengers. The only exception was CIP, due to the already mentioned more reactive neutral ionic state of this molecule at pH > 6.08 (see Section 3.1 and Section 3.4). Tap water could simulate better than MQ a post-treatment for specific water treatment and reuse goals [34,37].

The duty cycle (D) had a significant impact only on the *k_app_* of CIP. This result is in accordance with Figure 1, since CIP was the compound with the fastest reactivity towards ROS in the mixture, and the radical enhancement, caused by the lower duty cycle, should favor CIP degradation. The combined effect CD (catalyst presence and duty cycle) was also significant, given that any improvement related to lower duty cycles can only take place when the catalyst was used [38]. Controlled periodic illumination reduces charge careers recombination at the catalyst’s surface and can increase photocatalytic rates. The formation of ROS in the presence of light, in fact, takes place in femtoseconds (10^–15^ s), while their subsequential reactions with pollutants are much slower (6^–10^ s). Light does not have to be present in this slower stage for the reaction to be effective, so flicking the lights at high frequencies (500 Hz) improves the overall photonic efficiency of photocatalysis. A deeper explanation of the mechanisms of controlled periodic illumination can be found elsewhere [71,72]. The significance of the catalyst presence (C) was lower for CIP than for other PhACs, since CIP was the only compound degraded by UV-A photolysis alone.

The wavelength was the most significant effect for SMX, which is quickly degraded by UV-C photolysis alone. The matrix effects played a more significant role for SMX than for CIP, since the performance of UV-C is more sensitive to the changes in the matrix components and lower wavelengths are more easily absorbed [33]. SMX experiments confirm that different results can be obtained as a function of both the pollutant and the applied treatment.

TMP, VX, and DV reacted slowly at both photolytic and photocatalytic routes. The change in the matrix from MQ to tap water made the few agents capable of their degradation quickly unavailable and hindered the process considerably. Since TMP and VX did not degrade with photolysis alone, the presence of the catalyst was the most significant factor in increasing their degradation rates. For DV, the fastest degradation happened under lower wavelengths (UV-C).

**Figure 3:**
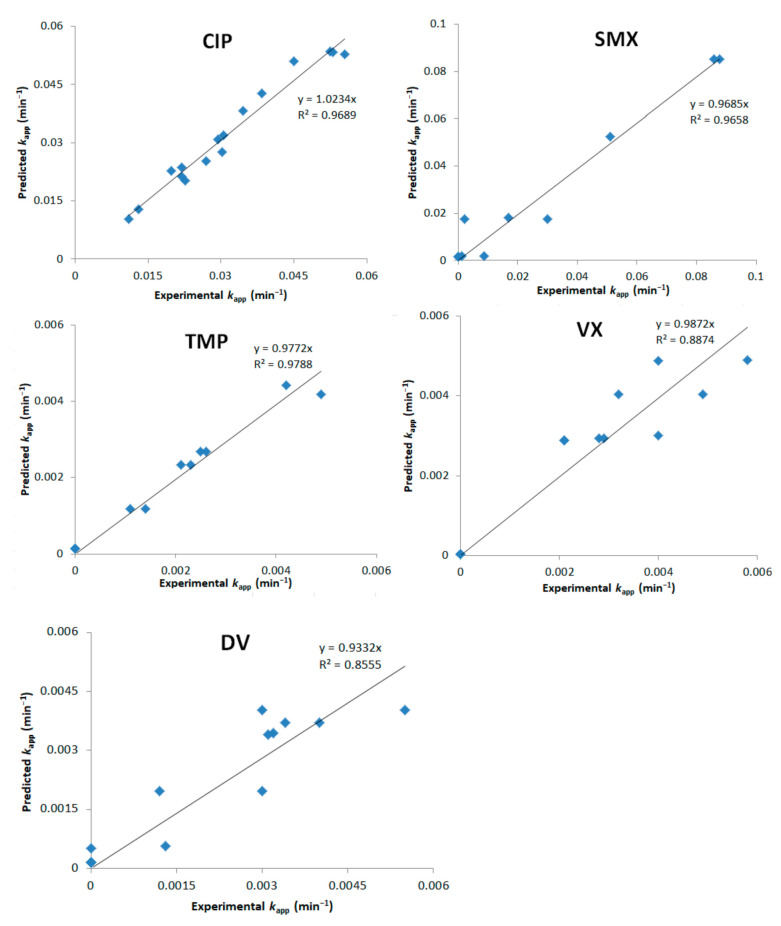
Model predictions vs. experimental *k_app_* values for the categorical DoE.

Plots comparing experimental *k_app_* values with the ones predicted by Equations (4)–(8) are shown in Figure 3. It can be observed that VX and DV did not have a correlation model as good as the other tested PhACs. Possible explanations are the influence of other significant factors in the degradation of these compounds, such as the presence of degradation products and pH, which were not evaluated in the DoE.

#### 3.2.2. Total Organic Carbon and Toxicity Analysis

**Figure 4:**
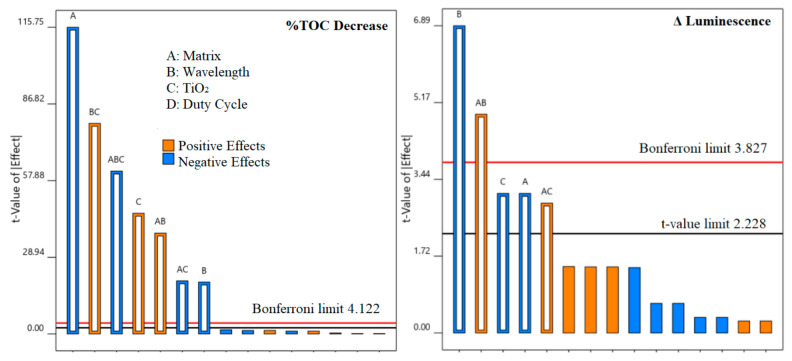
Pareto charts and significant effects (above Bonferroni limit) for %TOC decrease and Δ luminescence.

Since each compound may react differently, %TOC decrease can give a more comprehensive perspective of the degradation capability of the evaluated treatments. Equation (9) shows the obtained model from DoE. Figure 4 shows its correspondent Pareto chart.

(9) %TOC decrease=11.1187−6.1812A−1.0437B+2.4312C+2.0313AB−1.0688AC+4.2438BC−3.2813ABC

DoE analysis showed that the matrix (A) was the most significant effect in the Pareto chart for the overall degradation, expressed as %TOC decrease (Figure 4). Photocatalysis, like most AOPs, is highly dependent on matrix characteristics, and faster degradation is usually observed in MQ than in real matrices, which highlights the importance of pre-treatments for better performance [13]. Just like for individual PhACs, the BC (wavelength and catalyst presence) factor was highly significant and the highest mineralization rates were obtained with UV-A and a catalyst or UV-C without a catalyst. All effects involving duty cycle were not significant.

As regards toxicity, Equation (10) shows the obtained mathematical model, and its Pareto chart is also shown in Figure 4.

(10) Δ Luminescence=−0.6938−5.2188A−11.4938B−5.2188C+8.18125AB+4.8562AC

UV-C light was the main factor reducing toxicity. Conversely, the toxicity increase observed with UV-A can be credited, among other things, to transformation products of CIP that are more toxic than the parent compound [20]. The presence of the catalyst had a negative effect, contributing to the increase in the toxicity of the final effluent. A cleaner matrix (MQ water) was able to attain lower final toxicity levels because it allowed a faster degradation not only of the target compounds but also of degradation by-products, as demonstrated by the highest mineralization (TOC decrease) rates in MQ water.

**Figure 5:**
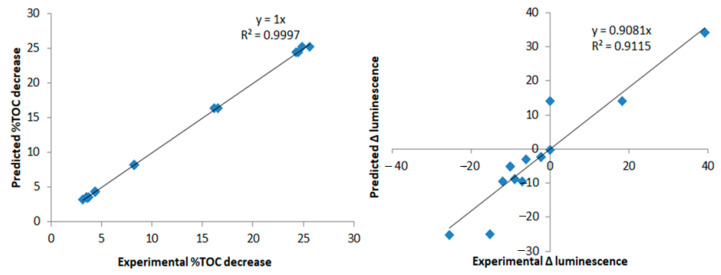
Model predictions vs. experimental %TOC decrease and Δ luminescence for the categorical DoE.

Figure 5 shows the model prediction for %TOC decrease and Δ luminescence, based on Equations (9) and (10) obtained from the design of experiments. %TOC decrease had an excellent fit. The prediction for luminescence had a lower accuracy but was still good, particularly considering how often prediction of toxicity involves much more complex and robust techniques, such as quantitative structure-activity relationship QSAR models [73].

### 3.3. Mechanism of Degradation

**Figure 6:**
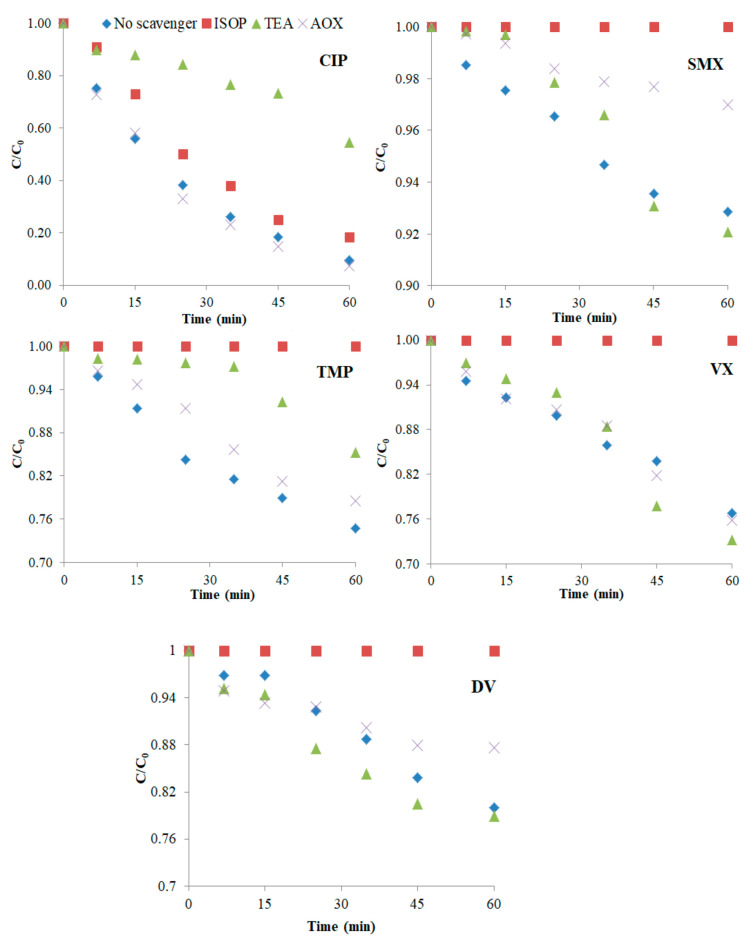
Degradation in the presence of scavengers (UV-A photocatalysis in MQ water).

**Figure 7:**
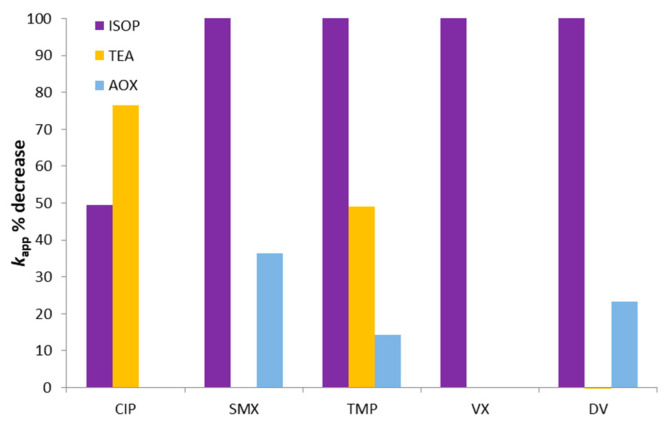
Effect of scavengers on PhACs *k_app_* values (UV-A photocatalysis in MQ water).

To investigate the UV-A photocatalytic degradation mechanisms, experiments with several radical scavengers were performed. Isopropanol (ISOP), triethanolamine (TEA), and ammonium oxalate (AOX) were adopted as scavengers of hydroxyl radicals (^•^OH), superoxide radicals (^•^O_2_^–^), and positive holes (*h*^+^), respectively [59,74,75]. Figure 6 shows the (*C*/*C*_0_) vs. time degradation plots of the PhACs (initial solution at a 100:1 scavenger/pollutant mass ratio) in MQ water after 60 min of degradation and Figure 7 shows the relative % decrease on *k_app_* values due to each scavenger.

The hydroxyl radical was the most substantial contributor to the degradation of the pollutants, since the addition of ISOP completely hampered the degradation of four PhACs and reduced the CIP degradation rate by 50%. However, CIP was even further inhibited by TEA, indicating a high reactivity of this pollutant with super oxide radicals as well. The contribution of holes to the degradation was smaller, being relevant only for SMX, DV, and TMP, but always below 40%. Attention should be paid to the fact that a severe inhibition of the degradation does not mean that the compound is directly highly reactive towards the respective scavenger. This becomes evident by evaluating VX, TMP, and DV degradation, which was completely halted by the presence of isopropanol; however, in the latter’s absence, the reaction rates were also very low (Figure 1). The pH range in which the reactions took place (pH_0_ = 6.7, pH_final_ = 5.7) can be a decisive parameter given that the amount of available radicals and holes depends on it [76].

### 3.4. Effect of the Initial pH

**Figure 8:**
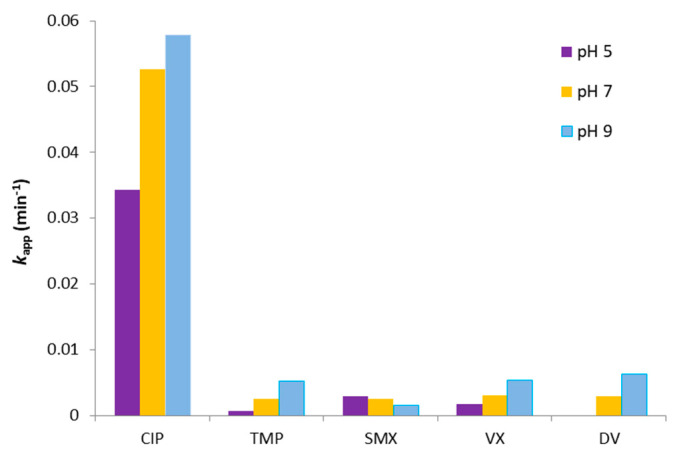
*k_app_* at different initial pH for the mixture of the five PhACs in MQ water, *C*_0_ = 2 mg/L each (UV-A photocatalysis).

Since matrix composition and pH are related, the effect of the latter in the degradation was also evaluated. The results in Figure 8 indicate that UV-A photocatalysis *k_app_* values were influenced by the initial pH, with most of the PhACs being depleted faster at higher pHs, which increased the production of reactive ROS species, such as ^•^OH [76].

The exception was SMX, which degraded faster at lower pHs. This was probably related to the more prominent role of the neutral (and more reactive) form of SMX at pH < 5.6 (SMX p*K*_a_ value) [65,69]. As was explained in Section 3.1, a CIP-predominant neutral ionic state tends to degrade faster at pH > 6.1. The same phenomenon can be observed for TMP at pH > 7.1. Since the p*K*_a_ values of VX and DV are above the range of the experiments, their different reaction rates can be explained mainly by the increase of ROS species production in more basic environments.

### 3.5. Effect of the Matrix Constituents: Bicarbonates, Nitrates, and Humic Acids

For a more in-depth study of the large influence of the matrix in photocatalytic processes (discussed in Section 3.2), another DoE was performed, investigating the impact of the concentrations of commonly found substances in water matrices. For this analysis, the Box–Behnken design was chosen because it provides surface response graphs which fit the continuous character of the chosen variables. For this DoE, three levels of each independent variable are established (see Table 2) and the method’s obtained mathematical module encompasses not only the chosen range limits (as the full factorial design of Section 3.2) but also the whole interval of this range. A deeper discussion of this method and its application can be found in [77].

The chosen substances were bicarbonates (HCO_3_^–^), nitrates (NO_3_^–^), and humic acids (HA). They are commonly found in waterbodies, so their mechanisms of reaction with ROS species should be well understood to optimize AOPs. The influence of bicarbonates on the performance of AOPs has been documented [26,28]. This ion acts as a scavenger, reacting with the hydroxyl radical and forming the carbonate radical, according to Equation (11) [78]. This may hinder the degradation processes because, compared to the hydroxyl radical, the carbonate radical has a reaction rate considerably slower with most substances [79].

(11) $$\mathrm{OH}+{\mathrm{HCO}}_{3}{}^{-}\to \cdot {\mathrm{CO}}_{3}{}^{-}+{\;H}_{2}O$$

The presence of nitrates and humic acids in the photocatalytic reaction medium may cause positive and negative effects on radical production, with every target compound responding differently to specific process conditions. Humic acids may cause screening effects and divert radicals from the target compounds and lower their availability (negative effect) or they can generate new radicals via photosensitization under UV light (positive effect) [27,33,80]. Nitrates can act as electron capturers on the catalyst valence band, hampering ROS production (negative effect), or undergo photolysis under UV light, creating more radicals (positive effect) [80,81]. Additionally, the reaction of humic acids and nitrates with PhAC degradation products may cause all sorts of inhibition or promotion effects.

Figure S5 shows the ANOVA analysis of variance. It was possible to attain a significant model for four out of five PhACs. Their obtained coded Equations (12)–(15) are:

(12) $$\sqrt{k_{app}{}_{\left(CIP\right)}}=0.1940-0.0253A-0.0008B+0.0027C-0.0006AB-0.0023AC-0.0131BC$$

(13) $$\begin{array}{ccccc} \sqrt{k_{app}{}_{\left(SMX\right)}}=0.0340 & -0.007A & -0.0015B+0.0043C-0.0053AB-0.0017AC-0.0006BC+0.0062A^{2}-0.002B^{2} & -0.0027C^{2} & \end{array}$$

(14) $$\begin{array}{ccccc} k_{app}{}_{\left(VX\right)}=0.012 & +0.004A & -0.0005B-0.0005C+0.00005AB-0.0004AC+0.0007BC-0.0059A^{2}-0.0002B^{2} & -0.00017C^{2} & \end{array}$$

(15) $$\begin{array}{ccccc} \sqrt{k_{app}{}_{\left(DV\right)}}=0.1154 & +0.0445A & +0.0048B+0.0063C-0.0022AB-0.0032AC-0.0072BC-0.0447A^{2}-0.009B^{2} & +0.0053C^{2} & \end{array}$$

A significant model for the degradation of TMP could not be obtained (Figure S4). Possible explanations are: (1) an overall very low reactivity of TMP with all the agents present in the photocatalytic system; (2) a larger range of the tested variables is required; (3) the impact of other factors which were not accounted in the DoE was more significant (e.g., pH, different TMP reactivity for different ionization states, presence of multiple degradation products); (4) simultaneous positive and negative effects of matrix composition on *k_app_*, as previously commented.

**Figure 9:**
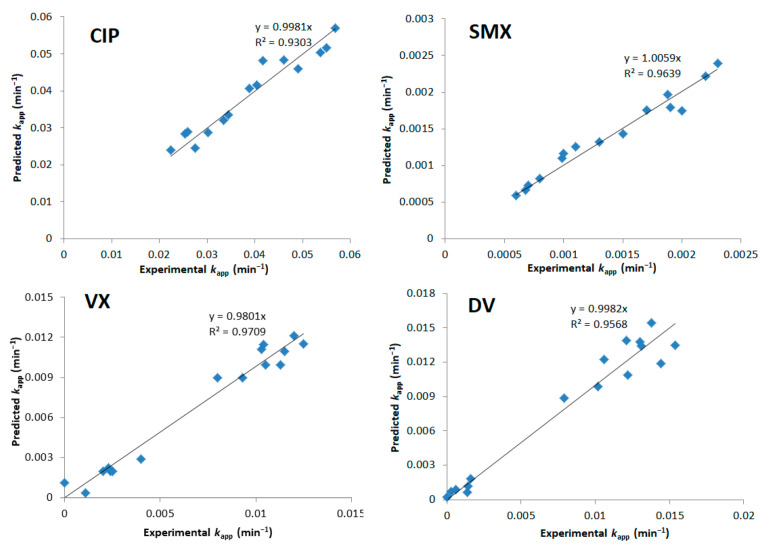
Predicted vs. experimental *k_app_* for CIP, SMX, VX, and DV (continuous surface DoE).

The experimental vs. the predicted (by Equations (11)–(14)) *k_app_* are shown in Figure 9, with good fits for all cases.

**Figure 10:**
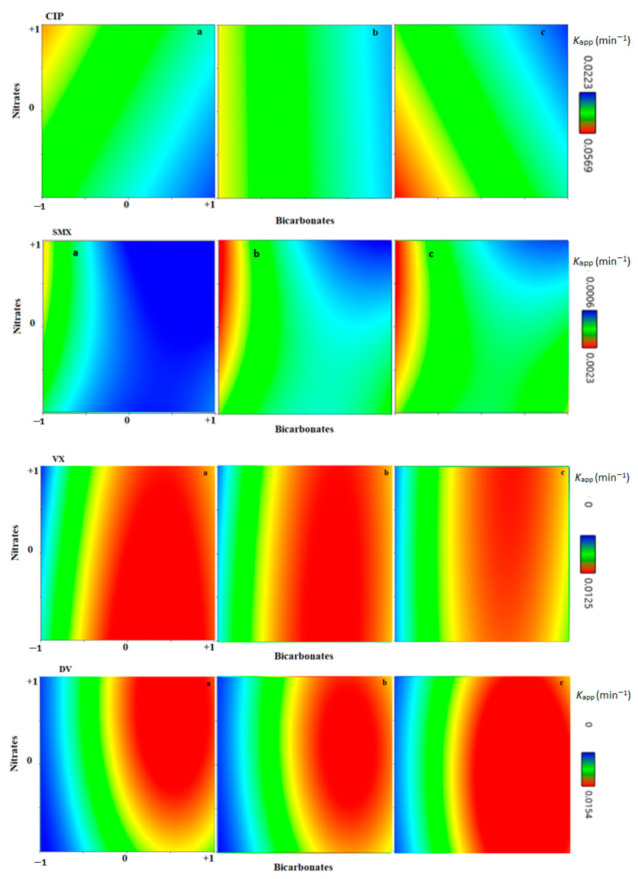
Surface graphs of *k_app_* for CIP, SMX, VX, and DV. X-axis: coded bicarbonates; Y-axis: coded nitrates; a, b, and c of each row represents humic acids coded values of −1, 0, and +1, respectively.

Surface graphs for CIP, SMX, VX, and DV are shown in Figure 10. High *F*-values were obtained for the coefficients A (bicarbonate) and A^2^ (when a quadratic fit was adopted), indicating their high significance. The addition of bicarbonate affected the initial pH of the solution, which in turn further supported the prominent significance of bicarbonate-related coefficients (Table S4). As shown in Figure 9 and the literature [16], the initial pH of the reaction impacts the photolytic degradation rate, the availability of radicals, the ionization state of each pollutant, and, consequently, the overall degradation rates. Since the initial pH and the bicarbonate concentrations are narrowly related, it is not possible to study them as independent variables. Thus, the addition of bicarbonates increases the complexity of the system, enabling a multitude of reaction pathways that take place simultaneously, some of them fostering a faster PhAC degradation rate, while others slow it down. In this study, CIP and SMX had their degradation hindered by bicarbonates, while both VX and DV had their *k_app_* increased by bicarbonate presence until a certain point.

The addition of nitrates and humic acids (B and C in Table 2, respectively) affected the *k_app_* of CIP and SMX. In the case of CIP, the significant combined effect BC shows that the presence of either nitrate (B) or HA (C) in the absence of the other was beneficial for CIP degradation. For SMX, the addition of humic acid had an overall positive effect, and the presence of nitrates was beneficial as long as bicarbonates were low (AB effect). For DV and VX, the influence of nitrates and humic acids was much smaller (Figure 10).

### 3.6. Electrical Energy per Order (EEO) Analysis

**Figure 11:**
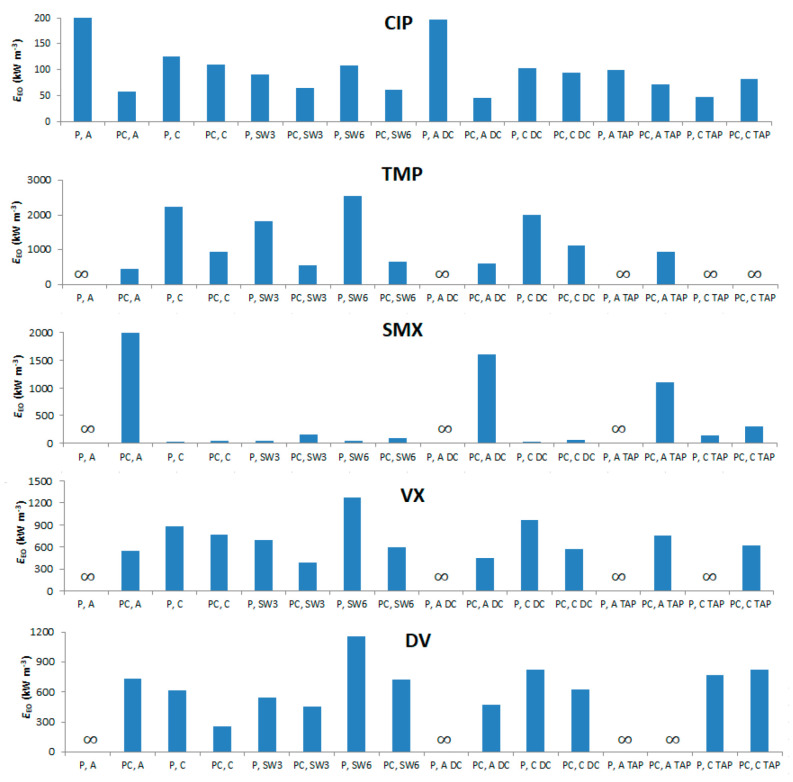
*E_EO_* values for the five PhACs for different processes. Experiments performed on MQ water, except the ones labelled with “tap” (tap water). Experiments performed under continuous illumination, except the ones labelled with “DC“ (duty cycle = 0.50).

It is fundamental to analyze the energy expenses of the photolytic and photocatalytic processes and how they are affected by variables, such as wavelength, matrix, and controlled periodic illumination. It was not possible to include *E_EO_* in the previous DoE analysis because all values corresponding to *k_app_* = 0 would tend to infinity. Figure 11 shows the *E_EO_* values of the five PhACs for different processes. The standard performances in MQ water were compared with cases using duty cycle and two varieties of simultaneous wavelength (SW). In the first one (P or PC, SW3 LEDs), two strips of UV-A LED and one of UV-C LED were used. In the second one (P or PC, SW6 LEDs), three strips of both UV-A and UV-C LEDs were used simultaneously. The objective was to analyze the possible benefit brought by additional light sources, controlled periodic illumination, and simultaneous wavelength on *E_EO_*, and to compare its results in MQ and tap water.

Studies have shown that controlled periodic illumination can be a powerful tool to reduce *E_EO_* [38], but care must be taken to consider the particularities of each system. Results show that the duty cycle had mixed results for energy consumption. Although CIP, SMX, VX, and DV reduced their *E_EO_*s, especially for the (PC, A) case, other processes had mixed results for all PhACs. Possibly, due to the small scale of the photoreactor, the addition of the ARDUINO control board added energy expenses that would be negligible at a larger scale. Another possibility is that the arbitrarily chosen duty cycle of 0.50 was not optimal in this case. Simultaneous wavelengths have shown interesting results for disinfection [82,83], but both cases with three and six LEDs did not present any significant gain in *E_EO_* compared to the standard photoreactor design. As explained elsewhere [33], the effect of wavelength coupling could benefit both photolysis and photocatalysis, but when light sources with too far away wavelengths (like the present case, UV-C and UV-A) are used simultaneously, no wave interference takes place. Adding more light sources (P, SW6 and PC, SW6) generally led to an increase in *E_EO_* values (except for photocatalysis of SMX). These results reinforce the conclusions of the previous work [37], in which it was shown that both CPI and the additional number of LED strips did not reduce *E_EO_* values of the target pollutant. Contrasting results were obtained with tap water, since some compounds had increasing *E_EO_* values for all cases (TMP and DV), while the others showed lower values for some processes. As was seen in Section 3.5, real water contains substances that can both increase or decrease degradation rates, depending on each situation and target compound.

Overall, the *E_EO_* values for photocatalysis obtained in this work are of the same order as the ones provided by a recent comprehensive literature review [33], mostly ranging between 100 and 1000 kWh m^−3^. Be that as it may, comparing *E_EO_* values of the five target PhACs in the recent literature results in largely different figures due to different process conditions (catalyst used, PhAC’s *C*_0_s, type of lamp, photoreactor design, matrix composition). To illustrate that, calculated *E_EO_* values of a study performed by [84] in hospital wastewaters using TiO_2_ P25 catalyst (slurry, 100 mg/L) and a solar lamp for VX, DV, and SMX degradation were 1920, 1900, and 2600 kWh m^−3^, respectively. In a study performed by [85], the photocatalytic degradation of CIP by Zn-doped Cu_2_O particles in ultrapure water illuminated by visible light (>400 nm) had a calculated *E_EO_* of 35,000 kWh m^−3^. Another study of photocatalytic degradation of CIP [86], using a Bi_2_Ti_2_O_7_/TiO_2_/RGO catalyst under visible light in ultrapure water had an *E_EO_* of 26,000 kWh m^−3^. Degradation of SMX and TMP in urban wastewater using a TiO_2_ P25 catalyst (slurry, 1000 mg/L) and UV-A LEDs obtained *E_EO_*s of 30 and 60 kWh m^−3^, respectively [87]. Lastly, degradation of TMP in ultrapure water was investigated by [88] using TiO_2_ and Ru/WO_3_/ZrO_2_ catalysts illuminated by a near UV–Vis lamp—both in solution and immobilized. The first catalyst attained EEO values of 15,300 and 21,500 kWh m^−3^ in solution and immobilized, respectively. The second catalyst obtained values of 9500 and 9000 kWh m^−3^ for solution and immobilized, respectively.

These values are still way beyond technology feasibility (<10 kWh m^−3^) [13] This, together with the potential formation of more toxic degradation by products (see Section 3.2), represents the main disadvantage of photocatalysis. Despite that, a non-significant difference was observed between UV-A and UV-C values. Until a few years ago, there were concerns that LEDs in the UV-C range were exceedingly energy-demanding when compared to longer wavelengths [35]. Certainly, each pollutant can have a different response depending on the emitted wavelength. Nevertheless, both UV-A and UV-C *E_EO_* magnitudes are on the same average level, which represents an important advance in this technology. UV-C can greatly contribute to the degradation of compounds highly reactive to it, e.g., sulfamethoxazole, which had the lowest *E_EO_* value of all the studied cases (28 kWh m^−3^).

The low photonic efficiency is still characteristic of photocatalysis itself [12], independently of the light source. Nevertheless, the recent developments in UV-LED technology could decrease *E_EO_* values 10 to 20 times in the next five years [2], which would have a direct impact on the feasibility and overall expenses of the process.

## 4. Practical Implications of this Study

After an exhaustive study investigating degradation of multiple compounds of emerging concern under different scenarios, electrical energy per order values of UV-LED photocatalysis are still way beyond feasibility values and real large-scale application of this technology is still distant. The quick development of UV-LEDs in the field of material sciences, which has been exponentially lowering this light source’s wall plug efficiency seems to be the main source of possible performance improvements, since up-scaling questions, such as mass transport of reactive species and UV light’s permeation through large reactors, remain as considerable technological hurdles.

In the pursuance of photocatalysis optimization, special attention should be paid to the kinetic response of the main target compounds. PhAC reactivity, in fact, varies drastically among tens of thousands of different substances and no single treatment or method could probably account for all of them. The optimal photoreactor design for each situation will depend on the full understanding of the matrix composition, the reactivity of target pollutants, and the required quality of the final effluent.

A potential advantage of UV-LED photocatalysis against other AOPs is the use of simultaneous wavelengths and the formation of multiple reactive species (^•^OH, ^•^O_2_^–^, and *h*^+^) which would open up more possibilities of reaction pathways to fulfill this difficult task of degrading thousands of vastly different pollutants. Short-term possible uses of UV-LED photocatalysis should focus on small scale point-of-use applications, as a final polishing step for drinking tap water, or adoption in places with difficult access to chemicals, such as O_2_ tanks and H_2_O_2_.

Moreover, there is a lack of information about the studied emerging compounds (hence their inclusion in the 2020/1161 Watch List). Any information regarding the possibility of their removal is hence of utmost importance. For future research, special attention should be paid to degradation products, since this work has shown that the toxicity of the effluent can increase after treatment, mainly depending on the chosen wavelength and the initial compounds in the matrix. It is important to monitor toxicity levels prior to and after photocatalysis to avoid this kind of issue.

## 5. Conclusions

In this work, the individual and simultaneous UV-LED degradation of five different pharmaceuticals from the current EU Watch List of substances of emerging concern (2020/1161) was studied under a wide variety of scenarios. A design of experiments approach involving multiple parameters was able to quantify the significance of different individual and combined effects on kinetic rate constants, effluents’ total organic carbon abatement, and toxicity levels.

The impact on the apparent first-order kinetic rate constant of initial pH, LEDs wavelength, presence of immobilized TiO_2_ catalyst, controlled periodic illumination, and matrix composition was investigated for each compound, and the obtained responses differed considerably depending on the target substance. Different ionic states, absorption spectra, degradation routes, and transformation products will impact the reactivity of each compound. The first categorical design of experiments showed that UV-A combined with TiO_2_ was the better option for faster degradation. UV-A alone, in fact, was incapable of degrading most of the pollutants and UV-C rays’ photolytic effects can be considerably hindered by the screening effect in the presence of a catalyst. The matrix also plays a large role in affecting the degradation rates. Higher pH values tend to favor faster UV-A photocatalytic degradation, but the influence of the ionic states with different reactivities should be carefully considered.

The mechanisms of reaction of each PhAC with ^•^OH, ^•^O_2_^–^, and *h*^+^ were also investigated, demonstrating the reactivity of the target compound to each of these species and showing that photocatalysis can degrade substances by different reaction routes. The second design of experiments focused on matrix composition using surface graphs and it showed that the presence of bicarbonates, humic acids, and nitrates can have mixed positive and negative effects on degradation.

Electrical energy per order values of UV-LED photocatalysis are still way beyond feasibility values. Controlled periodic illumination, simultaneous wavelength use, and additional lights did not considerably reduce energetic expenses. Nevertheless, UV-C LED values have now reached the same order of magnitude as UV-A, which was unattainable a few years ago. The potential near-future application of UV-LED photocatalysis that has been discussed involves small-scale point-of-use applications, and the formation of toxic degradation products demands special attention.

## Figures and Tables

**Table 1:** Independent coded variables corresponding to the first DoE.

Independent Coded Variables	−1	+1
Matrix (A)	MQ water	Tap water
LEDs wavelength (B)	272 nm (UV-C)	365 nm (UV-A)
TiO_2_ nanofilm presence (C)	No	Yes
Duty Cycle (D)	0.5	1.0 (continuous)

**Table 2:** Independent coded variables (quantities in mg/L) corresponding to the second DoE.

Independent Coded Variables	−1	0	+1
Bicarbonates (A)	0	200	400
Nitrates (B)	0	15	30
Humic acids (C)	0	1.5	3.0

## Data Availability

Not applicable.

## Supplementary Materials

The following supporting information can be downloaded at: https://www.mdpi.com/article/10.3390/nano12020295/s1, Figure S1: Experimental setup. 1—energy source, 2—power meter, 3—LED control board, 4—LED columns, 5—UV rays reaching the reactor, 6—reactor, and 7—magnetic stirrer; Figure S2: Upview of photoreactor with six UV-LED strips on; Figure S3: Degradation profiles for the individual degradation experiments; Figure S4: ANOVA results for categorical DoE by Design Expert 12 software; Figure S5: ANOVA results for surface response DoE by Design Expert 12 software; Table S1: Tap water composition in Zagreb, Croatia (pH = 7.4); Table S2: LED specifications; Table S3: Acronyms of the performed treatments; Table S4: Experiments performed (experiments 1–20: individual degradation; experiments 21–23: initial pH investigation; experiments 24–39: categoric design of experiments; exp 40–56: continuous surface response design of experiments; exp 57–60: simultaneous wavelengths).
