# Supplementary material for: Performance of TiO2/UV-LED-Based Processes for Degradation of Pharmaceuticals: Effect of Matrix Composition and Process Variables
Source: Nanomaterials (Basel). 2022 Jan 17;12(2):295. doi: 10.3390/nano12020295 (PMC8780436; doi:10.3390/nano12020295)
Supplement: Supplementary file 1 [file nanomaterials-12-00295-s001.zip › nanomaterials-1540656-supplementary.pdf]

nanomaterials

Supplementary Materials

Performance of TiO_2/UV-LED-Based Processes for Degradation of Pharmaceuticals: Effect of Matrix Composition and Process Variables

Danilo Bertagna Silva [1], Gianluigi Buttiglieri [2,3], Bruna Babić [1], Danijela Ašperger [1] and Sandra Babić [1,*]

[1] Faculty of Chemical Engineering and Technology, University of Zagreb, Trg Marka Marulića 19, 10000 Zagreb, Croatia; dsilva@fkit.hr (D.B.S.); bbabic@fkit.hr (B.B.); diva@fkit.hr (D.A.)
[2] Catalan Institute for Water Research (ICRA), C. Emili Grahit 101, 17003 Girona, Spain; gbuttiglieri@icra.cat
[3] University of Girona, Girona, Spain
* Correspondence: sandra.babic@fkit.hr

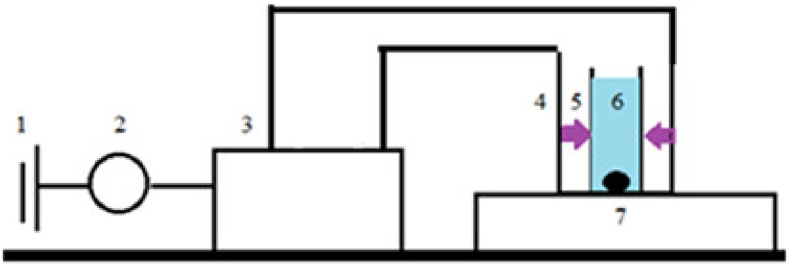

Figure S1. Experimental setup. 1 - energy source, 2 - power meter, 3 - LED control board, 4 - LED columns, 5 - UV rays reaching the reactor, 6 – reactor, and 7 - magnetic stirrer.

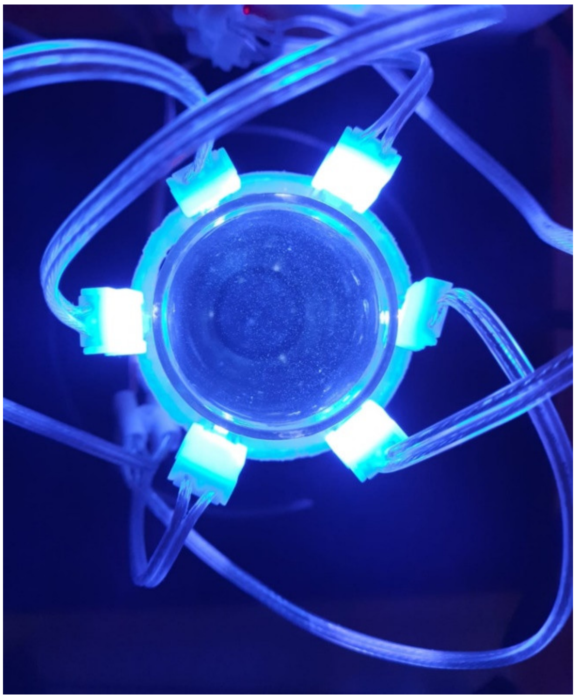

Figure S2. Upview of photoreactor with 6 UV-LED strips on.

Table S1. Tap water composition in Zagreb, Croatia (pH = 7.4).

Ions	mg/L
HCO_3^-	400
Cl^-	25
SO_4^{2-}	21
Br^-	<0.020
NO_3^-	15
NO_2^-	<0.020
PO_4^{3-}	<0.040
Na^+	13
NH_4^+	<0.042
Mg^{2+}	21
Ca^{2+}	95
TOC	<0.5

Table S2. LED specifications.

LED specification	Values	
	UV-A	UV-C
UV output per lamp	20 mW	2.5 mW
Emission angle	120°	120°
Input voltage	12 V	12 V
LED spacing	8.3 mm	16.6 mm
Wavelength	365 nm	272 nm
Spectrum FWHM	10 nm	10 nm
LED sources per strip	15	8

Table S3. Acronyms of the performed treatments.

TiO_2 nanofilm	LED strips	Acronym
No	3 UV-C	P, C
Yes	3 UV-C	PC, C
No	3 UV-A	P, A
Yes	3 UV-A	PC, A
No	1 UV-A, 2 UV-C	P, SW3
Yes	1 UV-A, 2 UV-C	PC, SW3
No	3 UV-A, 3 UV-C	P, SW6
Yes	3 UV-A, 3 UV-C	PC, SW6

Table S4. Experiments performed (experiments 1-20: individual degradation; experiments 21-23: initial pH investigation; experiments 24-39: categoric design of experiments; exp 40-56: continuous surface-response design of experiments; exp 57-60: simultaneous wavelengths).

	Initial concentration (mg/L)							Added (mg/L)						k_{app} (min^{-1})				
**Exp №**	**CIP**	**SMX**	**TMP**	**VX**	**MV**	**Treatment**	**Matrix**	**HCO_3^-**	**NO_3^-**	**Humic acids**	**Duty Cycle**	**Initial pH**	**Final pH**	**CIP**	**SMX**	**TMP**	**VX**	**DV**
1	2	0	0	0	0	P,A	MQ	0	0	0	1	6.0	5.4	0.0105				
2	0	2	0	0	0	P,A	MQ	0	0	0	1	5.7	5.7		0.0000			
3	0	0	2	0	0	P,A	MQ	0	0	0	1	6.4	6.3			0.0000		
4	0	0	0	2	0	P,A	MQ	0	0	0	1	5.8	5.7				0.0000	
5	0	0	0	0	2	P,A	MQ	0	0	0	1	7.0	6.7					0.0000
6	2	0	0	0	0	PC, A	MQ	0	0	0	1	6.0	4.8	0.0268				
7	0	2	0	0	0	PC, A	MQ	0	0	0	1	5.7	4.8		0.0038			
8	0	0	2	0	0	PC, A	MQ	0	0	0	1	6.4	5.6			0.0100		
9	0	0	0	2	0	PC, A	MQ	0	0	0	1	5.8	5.3				0.0097	
10	0	0	0	0	2	PC, A	MQ	0	0	0	1	7.0	5.9					0.0079
11	2	0	0	0	0	P,C	MQ	0	0	0	1	6.0	5.0	0.0220				
12	0	2	0	0	0	P,C	MQ	0	0	0	1	5.7	4.5		0.3663			
13	0	0	2	0	0	P,C	MQ	0	0	0	1	6.4	5.4			0.0000		
14	0	0	0	2	0	P,C	MQ	0	0	0	1	5.8	5.1				0.0027	
15	0	0	0	0	2	P,C	MQ	0	0	0	1	7.0	5.9					0.0094
16	2	0	0	0	0	PC, C	MQ	0	0	0	1	6.0	5.1	0.0244				
17	0	2	0	0	0	PC, C	MQ	0	0	0	1	5.7	4.7		0.1251			
18	0	0	2	0	0	PC, C	MQ	0	0	0	1	6.4	5.3			0.0076		
19	0	0	0	2	0	PC, C	MQ	0	0	0	1	5.8	5.1				0.0098	
20	0	0	0	0	2	PC, C	MQ	0	0	0	1	7.0	5.8					0.0112
21	2	2	2	2	2	PC,A	MQ	0	0	0	1	7.0	6.0	0.0526	0.0025	0.0026	0.0031	0.0030
22	2	2	2	2	2	PC,A	MQ	0	0	0	1	5.0	4.4	0.0343	0.0007	0.0030	0.0017	0.0000
23	2	2	2	2	2	PC,A	MQ	0	0	0	1	9.0	7.8	0.0578	0.0052	0.0015	0.0054	0.0063
24	2	2	2	2	2	P, A	MQ	0	0	0	1	6.7	6.3	0.0110	0.0000	0.0000	0.0000	0.0000
25	2	2	2	2	2	PC, A	MQ	0	0	0	1	6.7	5.8	0.0384	0.0012	0.0049	0.0040	0.0030
26	2	2	2	2	2	P, C	MQ	0	0	0	1	6.7	5.8	0.0198	0.0908	0.0000	0.0028	0.0034
27	2	2	2	2	2	PC, C	MQ	0	0	0	1	6.7	5.8	0.0226	0.0509	0.0034	0.0026	0.0064
28	2	2	2	2	2	P,A	MQ	0	0	0	0.5	6.7	6.3	0.0131	0.0000	0.0000	0.0000	0.0000

29	2	2	2	2	2	PC,A	MQ	0	0	0	0.5	6.7	5.7	0.0555	0.0016	0.0042	0.0058	0.0055
30	2	2	2	2	2	P,C	MQ	0	0	0	0.5	6.7	5.8	0.0271	0.0859	0.0014	0.0029	0.0034
31	2	2	2	2	2	PC,C	MQ	0	0	0	0.5	6.7	5.8	0.0294	0.0505	0.0025	0.0049	0.0045
32	2	2	2	2	2	P,A	TAP	0	0	0	1	7.7	7.6	0.0220	0.0000	0.0000	0.0000	0.0000
33	2	2	2	2	2	PC,A	TAP	0	0	0	1	7.7	7.6	0.0306	0.0020	0.0023	0.0029	0.0000
34	2	2	2	2	2	P,C	TAP	0	0	0	1	7.7	7.4	0.0525	0.0167	0.0000	0.0000	0.0032
35	2	2	2	2	2	PC,C	TAP	0	0	0	1	7.7	7.4	0.0302	0.0082	0.0000	0.0040	0.0030
36	2	2	2	2	2	P,A	TAP	0	0	0	0.5	7.7	7.6	0.0210	0.0000	0.0000	0.0000	0.0000
37	2	2	2	2	2	PC,A	TAP	0	0	0	0.5	7.7	7.6	0.0450	0.0024	0.0021	0.0029	0.0013
38	2	2	2	2	2	P,C	TAP	0	0	0	0.5	7.7	7.4	0.0530	0.0170	0.0000	0.0040	0.0030
39	2	2	2	2	2	PC,C	TAP	0	0	0	0.5	7.7	7.4	0.0345	0.0087	0.0000	0.0021	0.0012
40	2	2	2	2	2	PC,A	MQ	200	0	0	1	9.0	8.8	0.0335	0.0019	0.0007	0.0071	0.0079
41	2	2	2	2	2	PC,A	MQ	0	30	0	1	6.8	5.7	0.0550	0.0017	0.0019	0.0011	0.0006
42	2	2	2	2	2	PC,A	MQ	0	0	3	1	6.6	5.8	0.0569	0.0037	0.0017	0.0020	0.0014
43	2	2	2	2	2	PC,A	MQ	400	0	0	1	9.1	8.9	0.0275	0.0012	0.0008	0.0104	0.0102
44	2	2	2	2	2	PC,A	MQ	200	30	3	1	9,1	9.0	0.0223	0.0016	0.0007	0.0093	0.0106
45	2	2	2	2	2	PC,A	MQ	0	15	0	1	6.7	5.8	0.0490	0.0022	0.0020	0.0025	0.0014
46	2	2	2	2	2	PC,A	MQ	200	15	1.5	1	9.1	8.9	0.0302	0.0023	0.0010	0.0120	0.0131
47	2	2	2	2	2	PC,A	MQ	0	0	1.5	1	5.7	5.8	0.0461	0.0019	0.0019	0.0023	0.0000
48	2	2	2	2	2	PC,A	MQ	0	15	3	1	6.7	5.7	0.0538	0.0025	0.0022	0.0024	0.0016
49	2	2	2	2	2	PC,A	MQ	0	30	1.5	1	6.8	5.7	0.0417	0.0025	0.0023	0.0000	0.0003
50	2	2	2	2	2	PC,A	MQ	400	0	1.5	1	9.1	8.9	0.0259	0.0022	0.0015	0.0113	0.0122
51	2	2	2	2	2	PC,A	MQ	200	30	3	1	9.0	8.8	0.0345	0.0027	0.0011	0.0125	0.0154
52	2	2	2	2	2	PC,A	MQ	400	30	1.5	1	9.0	8.8	0.0304	0.0030	0.0006	0.0105	0.0144
53	2	2	2	2	2	PC,A	MQ	400	15	3	1	9.0	8.8	0.0302	0.0029	0.0013	0.0080	0.0138
54	2	2	2	2	2	PC,A	MQ	200	30	0	1	9.0	8.8	0.0404	0.0021	0.0006	0.0103	0.0130
55	2	2	2	2	2	PC,A	MQ	400	15	0	1	9.1	8.8	0.0254	0.0032	0.0019	0.0115	0.0121
56	2	2	2	2	2	PC,A	MQ	200	0	3	1	9.0	8.8	0.0371	0.0025	0.0010	0.0122	0.0147
57	2	2	2	2	2	P, SW3	MQ	0	0	0	1	6.7	6.0	0.0259	0.0589	0.0013	0.0034	0.0043
58	2	2	2	2	2	PC, SW3	MQ	0	0	0	1	6.7	5.6	0.0368	0.0157	0.0042	0.0061	0.0052
59	2	2	2	2	2	P, SW6	MQ	0	0	0	1	6.7	4.8	0.0373	0.0876	0.0016	0.0032	0.0035
60	2	2	2	2	2	PC, SW6	MQ	0	0	0	1	6.7	4.5	0.0659	0.0431	0.0063	0.0068	0.0056

Text S1: Kinetics of degradation

When photolysis and photocatalysis happen simultaneously, the concentration (*C*) of a target pollutant can be determined by:

$$-\frac{dC}{dt} = k_p \cdot C + k_{pc} \cdot C$$

in which k_p and k_{pc} are the kinetic constants for photolysis and photocatalysis, respectively. By integrating this equation in time, we obtain

$$-Ln\frac{C_{(t)}}{C_0} = (k_p + k_{pc}) \cdot t$$

The sum of k_p and $k_{pc}$is the apparent degradation rate k_{app}, so the global reaction can be described by a first order equation:

$$-Ln\frac{C_{(t)}}{C_0} = k_{app} \cdot t$$

Degradation profiles for the individual degradation (experiments 1 – 20 in **Table S4**) are shown on **Figure S4.** The k_{app} values shown in **Table S4** of supplementary information were all obtained with $R^2 > 0.95$, reinforcing that this model describes the reaction well.

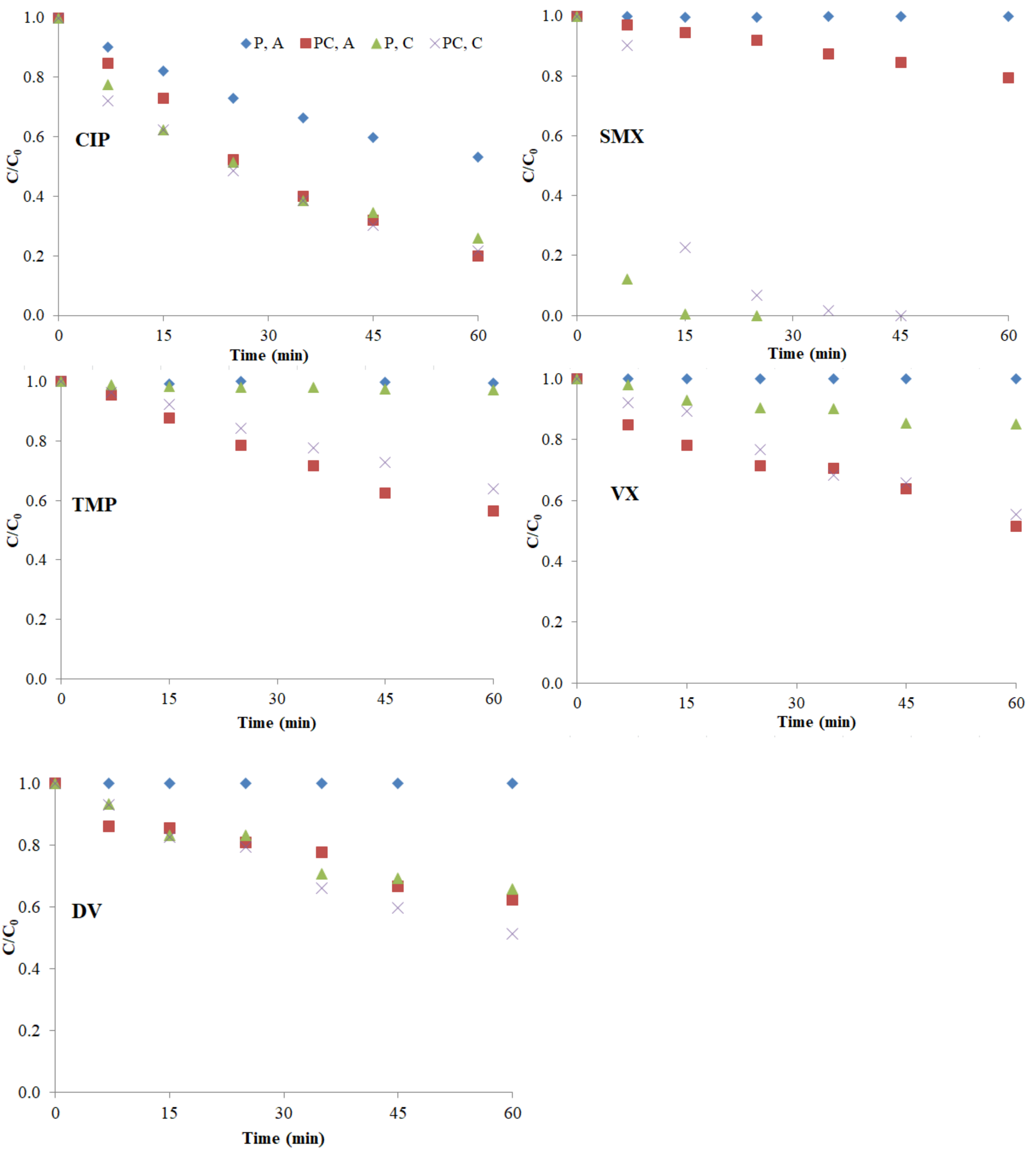

Figure S3. Degradation profiles for the individual degradation experiments.

Source	Sum of Squares	df	Mean Square	F-value	p-value	CIP
**Model**	0.0028	8	0.0004	32.58	< 0.0001	significant
A-Matrix	0.0003	1	0.0003	30.66	0.0009	
B-Wavelength	0.0001	1	0.0001	5.72	0.0480	
C-TiO_2	0.0003	1	0.0003	24.90	0.0016	
D-Duty Cycle	0.0002	1	0.0002	15.90	0.0053	
AB	0.0003	1	0.0003	28.03	0.0011	
AC	0.0004	1	0.0004	40.80	0.0004	
BC	0.0012	1	0.0012	108.44	< 0.0001	
CD	0.0001	1	0.0001	6.17	0.0420	
**Residual**	0.0001	7	0.0000			
**Cor Total**	0.0029	15				

Source	Sum of Squares	df	Mean Square	F-value	p-value	VX
**Model**	0.0135	7	0.0019	47.20	< 0.0001	significant
A-Matrix	0.0015	1	0.0015	37.34	0.0003	
B-Wavelength	0.0006	1	0.0006	13.94	0.0058	
C-TiO_2	0.0088	1	0.0088	216.99	< 0.0001	
AB	0.0005	1	0.0005	13.15	0.0067	
AC	0.0002	1	0.0002	5.09	0.0541	
BC	0.0009	1	0.0009	21.46	0.0017	
ABC	0.0009	1	0.0009	22.46	0.0015	
**Residual**	0.0003	8	0.0000			
**Cor Total**	0.0138	15				

Source	Sum of Squares	df	Mean Square	F-value	p-value	SMX
**Model**	0.0133	6	0.0022	45.59	< 0.0001	significant
A-Matrix	0.0024	1	0.0024	49.05	< 0.0001	
B-Wavelength	0.0053	1	0.0053	108.83	< 0.0001	
C-TiO2	0.0002	1	0.0002	3.79	0.0833	
AB	0.0040	1	0.0040	82.91	< 0.0001	
AC	0.0005	1	0.0005	9.27	0.0139	
BC	0.0010	1	0.0010	19.68	0.0016	
**Residual**	0.0004	9	0.0000			
**Cor Total**	0.0137	15				

Source	Sum of Squares	df	Mean Square	F-value	p-value	TMP
**Model**	0.0000	6	6.5×10^{-6}	99.34	< 0.0001	significant
A-Matrix	9.4×10^{-6}	1	9.5×10^{-6}	144.09	< 0.0001	
B-Wavelength	2.2×10^{-6}	1	2.2×10^{-6}	33.15	0.0003	
C-TiO_2	0.0000	1	0.0000	246.87	< 0.0001	
AB	5.3×10^{-7}	1	5.3×10^{-7}	8.01	0.0197	
AC	3.3×10^{-6}	1	3.3×10^{-6}	50.75	< 0.0001	
BC	7.4×10^{-6}	1	7.4×10^{-6}	113.15	< 0.0001	
**Residual**	5.9×10^{-7}	9	6.6×10^{-8}			
**Cor Total**	0.0000	15				

Source	Sum of Squares	df	Mean Square	F-value	p-value	DV
**Model**	0.0135	5	0.0027	17.26	0.0001	significant
A-Matrix	0.0020	1	0.0020	12.56	0.0053	
B-Wavelength	0.0065	1	0.0065	41.61	< 0.0001	
C-TiO_2	0.0021	1	0.0021	13.69	0.0041	
AC	0.0016	1	0.0016	10.06	0.0100	
BC	0.0013	1	0.0013	8.38	0.0160	
**Residual**	0.0016	10	0.0002			
**Cor Total**	0.0151	15				

Source	Sum of Squares	df	Mean Square	F-value	p-value	Δ Luminescence
**Model**	4433.49	5	886.70	19.91	< 0.0001	Significant
A-Matrix	435.77	1	435.77	9.78	0.0107	
B-Wavelength	2113.70	1	2113.70	47.46	< 0.0001	
C-TiO_2	435.77	1	435.77	9.78	0.0107	
AB	1070.93	1	1070.93	24.05	0.0006	
AC	377.33	1	377.33	8.47	0.0155	
**Residual**	445.36	10	44.54			
**Cor Total**	4878.85	15				

Source	Sum of Squares	df	Mean Square	F-value	p-value	%TOC decrease
**Model**	1268.04	7	181.15	3970.38	< 0.0001	significant
A-Matrix	611.33	1	611.33	13398.92	< 0.0001	
B-Wavelength	17.43	1	17.43	382.04	< 0.0001	
C-TiO_2	94.58	1	94.58	2072.89	< 0.0001	
AB	66.02	1	66.02	1446.92	< 0.0001	
AC	18.28	1	18.28	400.56	< 0.0001	
BC	288.15	1	288.15	6315.63	< 0.0001	
ABC	172.27	1	172.27	3775.68	< 0.0001	
**Residual**	0.3650	8	0.0456			
**Cor Total**	1268.40	15				

Figure S4. ANOVA results for categorical DoE by Design Expert 12 software.

Source	Sum of Squares	df	Mean Square	F-value	p-value	CIP
**Model**	0.0116	6	0.0019	21.37	< 0.0001	significant
A-Bicarbonates	0.0070	1	0.0070	76.67	< 0.0001	
B-Nitrates	5.9 x 10^{-6}	1	5.9 x 10^{-6}	0.0647	0.8049	
C-Humic acid	0.0001	1	0.0001	0.7926	0.3965	
AB	2.8 x 10^{-6}	1	2.8 x 10^{-6}	0.0309	0.8645	
AC	0.0000	1	0.0000	0.4399	0.5238	
BC	0.0011	1	0.0011	11.66	0.0077	
**Residual**	0.0008	9	0.0001			
**Cor Total**	0.0125	15				

Source	Sum of Squares	df	Mean Square	F-value	p-value	TMP
**Model**	3.2 x 10^{-6}	6	5.3 x 10^{-7}	1.65	0.2394	not significant
A-Bicarbonates	1.2 x 10^{-9}	1	1.2 x 10^{-9}	0.0037	0.9530	
B-Nitrates	2.0 x 10^{-8}	1	2.0 x 10^{-8}	0.06	0.8059	
C-Humic acid	3.4 x 10^{-7}	1	3.4 x 10^{-7}	1.07	0.3279	
AB	5.7 x 10^{-7}	1	5.7 x 10^{-7}	1.80	0.2128	
AC	5.9 x 10^{-7}	1	5.9 x 10^{-7}	1.84	0.2083	
BC	6.9 x 10^{-7}	1	6.9 x 10^{-7}	2.15	0.1768	
**Residual**	2.9 x 10^{-6}	9	3.2 x 10^{-7}			
**Cor Total**	6.0 x 10^{-6}	15				

Source	Sum of Squares	df	Mean Square	F-value	p-value	SMX
**Model**	0.0010	9	0.0001	21.96	0.0006	significant
A-Bicarbonates	0.0005	1	0.0005	90.20	< 0.0001	
B-Nitrates	0.0000	1	0.0000	3.40	0.1148	
C-Humic acid	0.0001	1	0.0001	23.13	0.0030	
AB	0.0002	1	0.0002	36.62	0.0009	
AC	0.0000	1	0.0000	2.77	0.1473	
BC	2.2 x 10^{-6}	1	2.2 x 10^{-6}	0.4350	0.5340	
A^2	0.0001	1	0.0001	18.56	0.0050	
B^2	0.0000	1	0.0000	1.98	0.2095	
C^2	0.0000	1	0.0000	4.55	0.0768	
**Residual**	0.0000	6	5.0 x 10^{-6}			
**Cor Total**	0.0010	15				

Source	Sum of Squares	df	Mean Square	F-value	p-value	VX
**Model**	0.0003	9	0.0000	22.63	0.0006	significant
A-Bicarbonates	0.0002	1	0.0002	111.08	< 0.0001	
B-Nitrates	1.6 x 10^{-6}	1	1.6 x 10^{-6}	1.05	0.3460	
C-Humic acid	2.5 x 10^{-6}	1	2.5 x 10^{-6}	1.64	0.2475	
AB	1.6 x 10^{-6}	1	1.6 x 10^{-6}	1.07	0.3403	
AC	1.2 x 10^{-6}	1	1.2 x 10^{-6}	0.77	0.4149	
BC	2.9 x 10^{-6}	1	2.9 x 10^{-6}	1.93	0.2146	
A^2	0.0001	1	0.0001	48.33	0.0004	
B^2	1.5 x 10^{-6}	1	1.5 x 10^{-6}	0.0978	0.7651	
C^2	6.2 x 10^{-6}	1	6.2 x 10^{-6}	0.0412	0.8459	
**Residual**	9.0 x 10^{-6}	6	1.5 x 10^{-6}			
**Cor Total**	0.0003	15				

Source	Sum of Squares	df	Mean Square	F-value	p-value	DV
**Model**	0.0279	9	0.0031	21.90	0.0006	significant
A-Bicarbonates	0.0187	1	0.0187	131.81	< 0.0001	
B-Nitrates	0.0002	1	0.0002	1.30	0.2984	
C-Humic acid	0.0003	1	0.0003	2.25	0.1841	
AB	0.0000	1	0.0000	0.23	0.6518	
AC	0.0001	1	0.0001	0.45	0.5261	
BC	0.0003	1	0.0003	1.78	0.2310	
A^2	0.0053	1	0.0053	37.67	0.0009	
B^2	0.0003	1	0.0003	1.87	0.2202	
C^2	0.0001	1	0.0001	0.64	0.4550	
**Residual**	0.0008	6	0.0001			
**Cor Total**	0.0288	15				

Figure S5. ANOVA results for surface-response DoE by Design Expert 12 software.
